# A Linear {Fe^III^_2_Ni^II^} Cluster, a Structurally Closely Related *Quasi* 1D {Fe^III^_2_Ni^II^} Chain

**DOI:** 10.1002/chem.202500911

**Published:** 2025-05-28

**Authors:** Emmanouil K. Charkiolakis, David Gracia, Marc Ubach I Cervera, Marco Evangelisti, Constantinos J. Milios

**Affiliations:** ^1^ Department of Chemistry The University of Crete Voutes Herakleion 71003 Greece; ^2^ Instituto de Nanociencia y Materiales de Aragón (INMA) CSIC Universidad de Zaragoza C/ Pedro Cerbuna, 12 Zaragoza 50009 Spain

**Keywords:** coordination polymers, heat capacity, heterometallic Fe^III^/Ni^II^ species, magnetic properties, trimetallic complexes

## Abstract

The solvothermal reaction between FeCl_3_·6H_2_O, NiCl_2_·6H_2_O, 2‐hydroxynaphthaldehyde, and glycine (gly) in MeOH results in the formation of the trinuclear heterometallic cluster [Fe^ΙΙΙ^_2_Ni^ΙΙ^(L_1_)_4_(MeOH)_4_] (**1**) in good yield, while the same reaction upon replacing gly with methyl‐alanine (mAla), results in the formation of the closely related quasi 1D coordination polymer {[Fe^ΙΙΙ^_2_Ni^ΙΙ^(L_2_)_4_(MeOH)_2_]_n_} (**2**) (L_1_, L_2_: the dianionic form of the Schiff‐base ligand derived from the condensation of 2‐hydroxynaphthaldehyde and gly (L_1_ for **1**), or mAla (L_2_ for **2**)). The structure of **1** describes a linear trimetallic {Fe^III^_2_Ni} unit, whereas complex **2** consists of repeating linear {Fe^III^_2_Ni} units with each one connected to two neighboring units via four coordination bonds, forming a *quasi*‐1D chain. The structural similarity between complex **1** and the repeating unit of complex **2** is remarkable. However, their magnetic properties differ significantly, with complex **1** displaying dominant ferromagnetic interactions, and complex **2** showing antiferromagnetic interactions.

## Graphical abstract

The synthesis, characterization, and magnetic properties of two closely related heterometallic {Fe^III^_2_Ni^II^} species are reported, upon employment of suitable amino acid Schiff‐base ligands; a discrete {Fe^III^_2_Ni^II^} trimetallic cluster and a {Fe^III^_2_Ni^ΙΙ^}_n_ chain. Despite their striking structural similarity, they display contrasting magnetic properties, with the former showing ferromagnetic and the latter antiferromagnetic behavior.

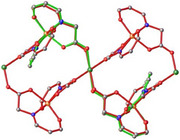

## 1 Introduction

The study of coordination polymers has attracted considerable attention in recent years due to their unique structural features and potential applications in various fields, including materials science, catalysis, and magnetism.^[^ ^1^ ^]^ Such species often adopt diverse architectures that exhibit interesting physical and chemical properties. Furthermore, transition metal coordination polymers based on paramagnetic 3*d* metallic centers, are of particular interest due to their rich magnetic behavior, which arises from the interplay between the metal ions’ electronic configurations and their geometric arrangements/properties as dictated by the ligands’ sphere.^[^ ^2^ ^]^ Understanding the magnetic interactions and dynamics in such polymeric species is not only of fundamental importance for the field of molecular magnetism, but also opens new directions toward technological applications in magnetic devices and materials. Tailoring magnetic properties at the molecular level enables the development of high‐performance materials for spintronic applications, in which electron spin, rather than charge, is utilized for information processing. Such materials present great potential for next‐generation data storage technologies, offering higher capacity and energy‐efficient solutions. Moreover, coordination polymers of paramagnetic metal centers are being explored for biomedical applications, such as targeted drug‐delivery and hyperthermia therapy, where external magnetic fields can precisely regulate therapeutic responses. Therefore, it becomes evident that the synergy between molecular design and magnetic functionality places these materials at the cutting edge of interdisciplinary research, bridging chemistry, physics, and materials science.^[^ ^3^, ^4^ ^]^

Detailed studies on magnetically interesting coordination polymers have highlighted the importance of the ligands’ nature and the metal‐to‐ligand ratio in determining the resultant magnetic properties.^[^ ^5^ ^]^ For instance, complexes featuring Schiff‐base ligands have shown magnetic exchange interactions between neighboring metal centers, ranging from ferro‐ to antiferromagnetic, depending on the specific coordination environment and the type/nature of ligand.

We previously reported the synthesis and full characterization of a ribbon‐like 1D heterometallic {Cr^III^_2_Ni^II^} coordination polymer, upon employment of the Schiff‐base ligand derived from 2‐hydroxynaphthaldehyde and 2‐amino‐isobutyric acid, which was found to display slow relaxation of magnetization with a high relaxation energy barrier of ∼143 K above *T*c = 0.6 K.^[^ ^6^ ^]^ We herein, extend our studies to Fe^III^/Ni^II^ chemistry, and we present the synthesis and characterization of two closely related species, upon switching the amino acid part of the Schiff‐base ligand; a {Fe^III^_2_Ni^II^} linear trimetallic cluster with the employment of gly and its 1D {Fe^III^_2_Ni^II^}_n_ “analog” with the use of mAla.

## 2 Results and Discussion

### 2.1 Syntheses

The solvothermal reaction between FeCl_3_·6H_2_O, NiCl_2_·6H_2_O, 2‐hydroxynaphthaldehyde, and gly, in MeOH, under basic conditions, led to the formation of the trinuclear heterometallic complex [Fe^ΙΙΙ^_2_Ni^ΙΙ^(L_1_)_4_(MeOH)_4_] (**1**) (L_1_: the dianionic form of the Schiff‐base ligand between 2‐hydroxynaphthaldehyde and gly), in good yield, according to the following chemical Equation (1):

(1) $$\begin{array}{cc} & 2\;\mathrm{FeC}l_{3}\cdot 6H_{2}O+4\mathrm{NiC}l_{2}\cdot 6H_{2}O+4\;\mathrm{OH}\text{-naphth}+4\;\mathrm{gly}\\ & \to \left[F{e^{\mathrm{III}}}_{2}Ni^{\mathrm{II}}{\left(L_{1}\right)}_{4}{\left(\mathrm{MeOH}\right)}_{4}\right]+8\;H^{+}+8\;Cl^{-}+22\;H_{2}O \end{array}$$

The crystal structure of the complex (*vide* infra) revealed the formation of a linear trimetallic {Fe^III^_2_Ni^II^} cluster, assembled by the presence of the dianion of the Schiff‐base ligand that forms in‐situ from the reaction between 2‐hydroxynaphthaldehyde and gly. The initial Fe:Ni:“Schiff‐base” ligand ratio used was 2:1:4, which is exactly the ratio found in complex **1**, while upon changing various synthetic parameters, for example, the metal‐to‐ligand ratio and the nature of the base, led to the same product, as shown by pXRD comparison, *albeit* in lower yields. Finally, the same reaction under standard temperature and pressure laboratory conditions, led to the formation of an amorphous precipitate, which was not further analyzed.

With the identity of complex **1** confirmed, the next step was to investigate whether the nature of the Schiff‐base ligand would influence the product isolated. To investigate this, we replaced the natural amino acid gly used in the synthesis of **1** with the artificial amino acid mAla, and from the solvothermal reaction between FeCl_3_·6H_2_O, NiCl_2_·6H_2_O, 2‐hydroxynaphthaldehyde and mAla in MeOH, in the presence of CH_3_ONa, we successfully synthesized and isolated the 1D heterometallic coordination polymer {[Fe^ΙΙΙ^_2_Ni^ΙΙ^(L_2_)_4_(MeOH)_2_]_n_} (**2**) (L_2_: the dianionic form of the Schiff‐base ligand between 2‐hydroxynaphthaldehyde and mAla), according to the following stoichiometric chemical Equation (2):

(2) $$\begin{array}{cc} & 2n\;\mathrm{FeC}l_{3}\cdot 6H_{2}O+4n\;\mathrm{NiC}l_{2}\cdot 6H_{2}O+4n\;\text{OH-naphth}+4n\;\mathrm{mAla}\\ & \to \left\{{\left[F{e^{\mathrm{III}}}_{2}Ni^{\mathrm{II}}{\left(L_{2}\right)}_{4}{\left(\mathrm{MeOH}\right)}_{2}\right]}_{n}\right\}+8n\;H^{+}+8n\;Cl^{-}+22n\;H_{2}O \end{array}$$

The crystal structure of the complex (*vide infra*) revealed that **2** is a 1D coordination polymer consisting of repeating trimetallic {Fe^III^_2_Ni^II^} units that present striking structural similarity to complex **1**; however, complex **2** adopts a 1D polymeric architecture, while complex **1** exists as a discrete trimetallic cluster. As in the case of **1**, repeating the reaction under ambient temperature and pressure conditions afforded a brownish amorphous precipitate that was not further analyzed.

### 2.2 Description of Structures

**Figure 1:**
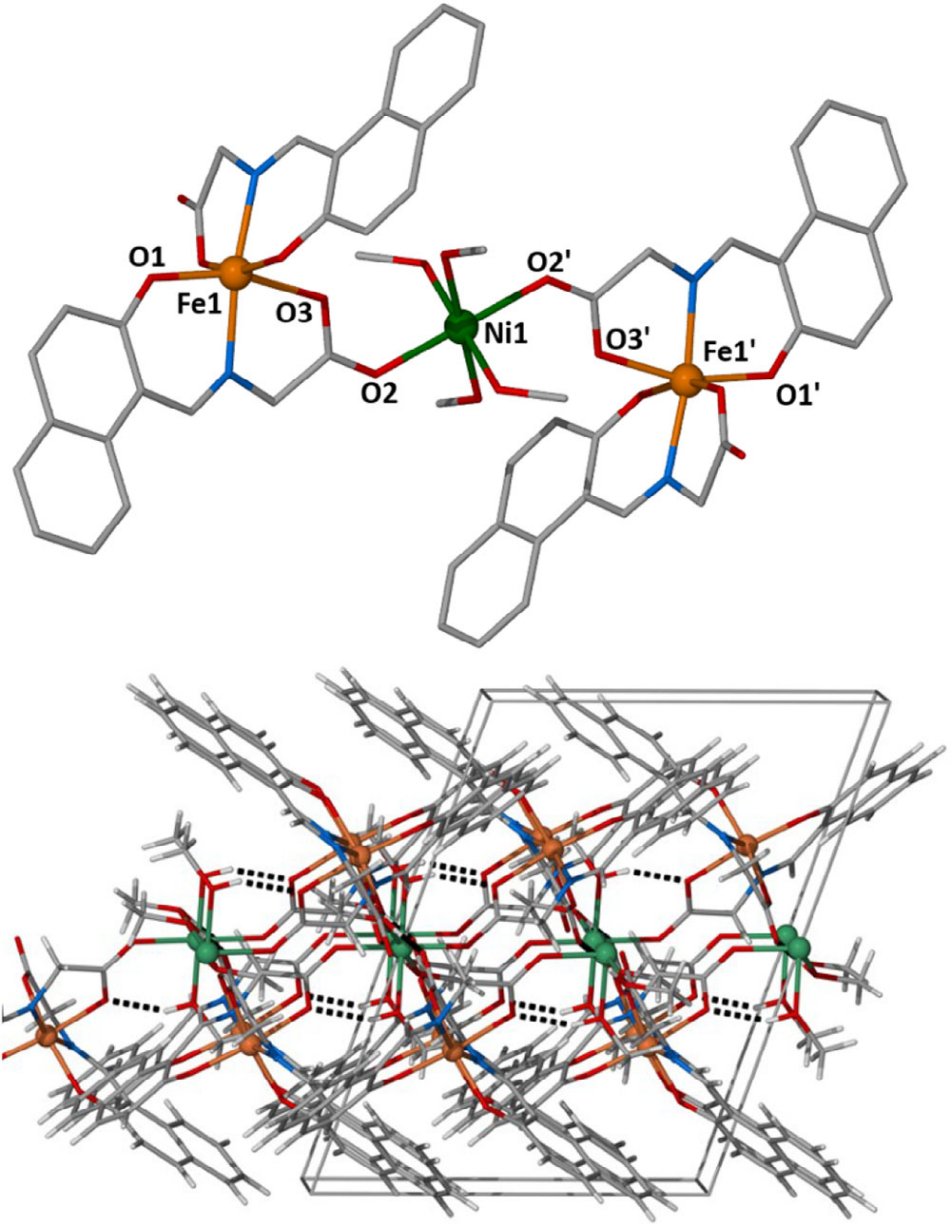
Top) The crystal structure of complex **1**. Bottom) The crystal packing of **1** highlighting the intra‐ and inter‐molecular H‐bonds. Color code: Fe^III^ = orange, Ni^II^ = green, O = red, N = blue, C = grey, and H = white.

Complex **1** crystallizes in the monoclinic space group *P*2_1_/*c* and its structure describes a linear trimetallic {Fe^III^_2_Ni^II^} assembly (Figure 1, top). The central Ni(II) atom is located ∼5.4 Å from the terminal ferric atoms, with each Fe(III) center connected to the central nickel atom via a *syn, anti η* ^1^:*η* ^1^:*µ* carboxylate group belonging to a fully deprotonated L_1_^2‐^ ligand. The coordination environment on the Fe(III) center is occupied by two fully deprotonated L_1_^2−^ ligands found in an *η* ^1^:*η* ^1^:*η* ^1^:*η* ^1^:*µ* manner, forming two six‐member and two five‐member chelate rings around the ferric metal ion, in an {O_4_N_2_} sphere. All metallic centers are six‐coordinate and adopt octahedral geometry, with the central nickel atom completing its coordination sphere with four terminal MeOH molecules, resulting in an {O_6_} environment. In the crystal lattice, molecules of **1** stack upon each other in a *zig‐zag* fashion, while the crystal lattice is stabilized by intra‐ and inter‐molecular hydrogen bonds (Figure 1, bottom, and Figure S1). Regarding the former H‐bonds, these occur between two MeOH molecules coordinated on the central nickel atom and two O_carboxylate_ belonging to fully deprotonated L_1_^2‐^ ligands, one at each side of the central Ni(II) [Ο7‐Η7^…^Ο3, Η^…^Ο 1.89 Å, Ο^…^Ο 2.651 Å, Ο‐Η^…^Ο 153^ο^]. As far as the intermolecular H‐bonds are concerned, each {Fe^III^_2_Ni^II^} cluster forms four H‐bonds to four neighboring {Fe^III^_2_Ni^II^} clusters, via the remaining two coordinated MeOH molecules on the central Ni(II) atom and two O_carboxylate_ belonging to fully deprotonated L_1_^2‐^ ligands [Ο8‐Η8^…^Ο6, Η^…^Ο 1.80 Å, Ο^…^Ο 2.590 Å, Ο‐Η^…^Ο 159^ο^].

**Figure 2:**
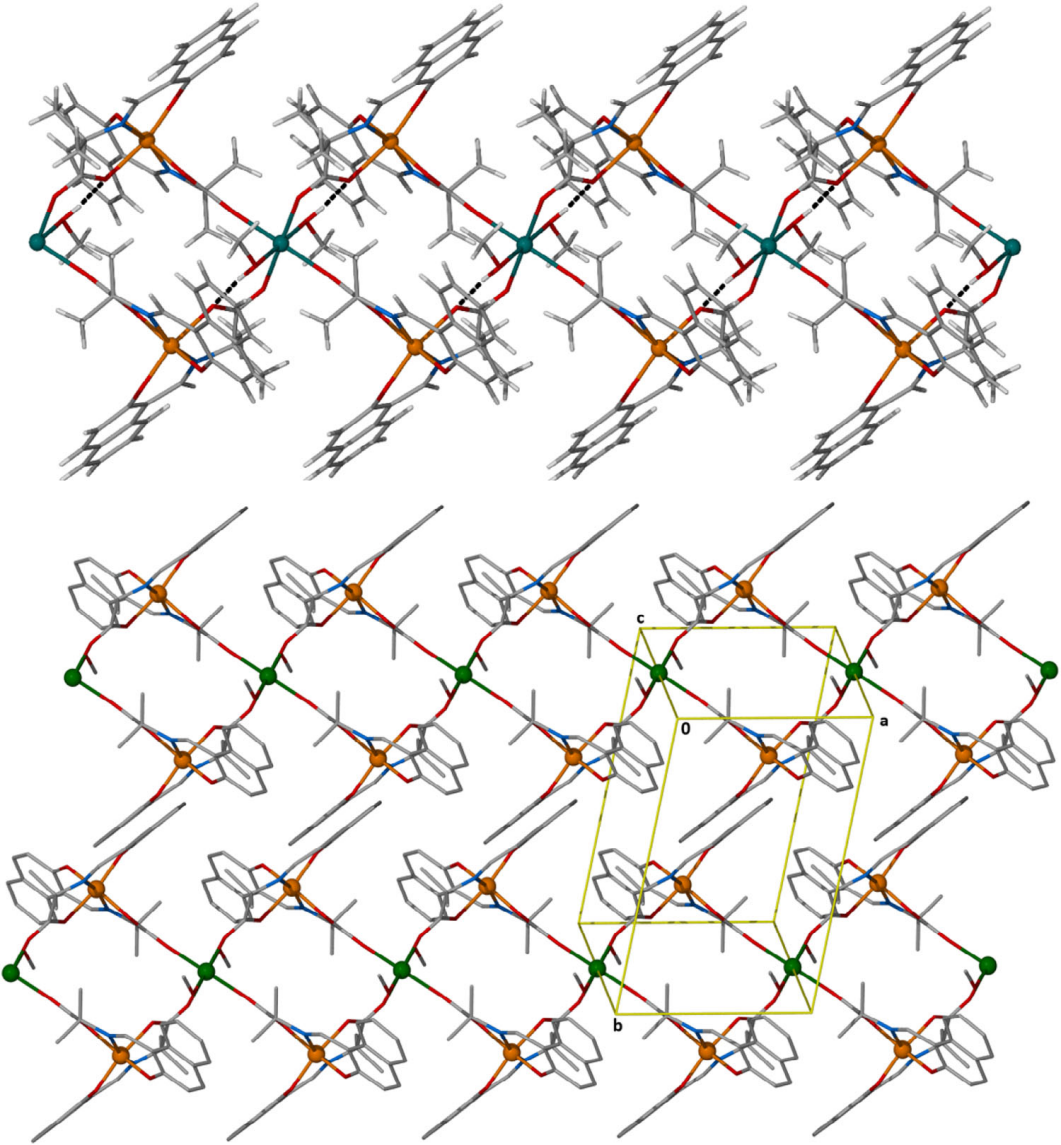
Top) The crystal structure of complex **2,** highlighting the H‐bonds (dotted black lines). Bottom) The arrangement of the ribbons of **2** in the crystal lattice. Color code: Fe^III^ = orange, Ni^II^ = green, O = red, N = blue, C = gray, and H = white.

Complex **2** crystallizes in the triclinic space group *P‐*1. Its repeating unit consists of linear [Fe^III^_2_Ni^II^(L_1_)_4_(MeOH)_2_] species, which are interconnected through four coordination bonds with neighboring units (Figure 2, top). Within each trimetallic unit, the central Ni^II^ atom is located ∼5.4 Å from the terminal Fe(III) atoms. Each iron center is connected to the central nickel center via a *syn, anti η* ^1^:*η* ^1^:*µ* carboxylate group belonging to a fully deprotonated L_2_^2−^ ligand. On each Fe(III), two fully deprotonated L_2_^2−^ ligands are bound in an *η* ^1^:*η* ^1^:*η* ^1^:*η* ^1^:*µ* fashion, forming two six‐member and two five‐member chelate rings around the metal ion. Furthermore, one L_2_^2−^ ligand provides the intra‐trimetallic linkage, while the remaining one connects neighboring trimetallic units via *anti, anti η* ^1^:*η* ^1^:*µ* carboxylate bonding. All metallic centers are six‐coordinate adopting octahedral geometry, with the central nickel atom completing its coordination sphere with two terminal MeOH molecules. The assembly of the trimetallic repeating units results in a ribbon‐like quasi‐1D coordination polymer that propagates parallel to the a‐axis, with the mean plane of the ribbon aligned parallel to the *ac* plane, providing coplanarity among all metallic centers (Figure 2, bottom). Alternatively, the ribbon‐like polymer can be described as corner‐sharing {Fe_2_Ni_2_} rhombuses, with intra‐ribbon Fe^…^Fe and Ni^…^Ni distances of ∼ 7.4 Å and 8.9 Å, respectively, and Fe^…^Ni distances of ∼6.1 Å and 5.4 Å. Within each ribbon, there are two H‐bonds present, between the two MeOH molecules on the central nickel atom and two O_carboxylate_ belonging to fully deprotonated L_2_^2‐^ ligands, one at each side of the central Ni(II) [Ο7‐Η7^…^Ο5, Η^…^Ο 1.88 Å, Ο^…^Ο 2.691 Å, Ο‐Η^…^Ο 162^ο^], while no intermolecular H‐bonds are present in the crystal lattice. In the crystal lattice, the ribbons form a 2D layer parallel to the *ac* plane, with the mean metallic planes of the closest intra‐layer ribbons spaced at ∼2.4 Å apart, and nearest inter‐ribbon metallic distances of ∼8.0 Å.

**Figure 3:**
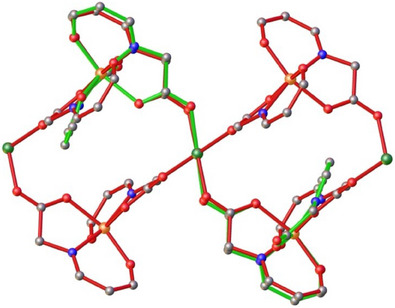
Overlay of **1** (green fragment) and **2** (red fragment) showing only the connectivity patterns. Atoms’ color code: Fe^III^ = orange, Ni^II^ = dark green, O = red, N = blue, C = gray. Aromatic rings, ‐CH_3_ groups, MeOH molecules, and H‐atoms are omitted for clarity.

A close inspection/comparison between the crystal structures of **1** and the repeating unit of **2** reveals that both trimetallic species display near identical structural parameters; this similarity is clearly demonstrated in the overlay diagram of their structures (Figure 3), where only minimal deviation between the species is observed. Taking into account that: i) the two Schiff‐base ligands, H_2_L_1_ and H_2_L_2_, differ only in the structure of the amino acid employed, gly in **1** versus mAla in **2**, ii) both gly and mAla display essentially the same pK_a_ values (2.35 for gly vs. 2.36 for mAla), and iii) the same reactions’ conditions, it becomes apparent that the change from 0‐ to 1‐D (from **1** to **2**) should be attributed to the structure of the amino acid ligands, imposing different steric hindrance effects and/or very weak intermolecular interactions.

The purity of the bulk samples was verified by means of: i) energy dispersive X‐ray spectroscopy (EDS) on **1** and **2**, that provided excellent agreement of the Fe^III^/Ni^II^ ratios (with the theoretical value of 2:1 as determined by the single‐crystal structure determination (Figure S2), and ii) pXRD diagrams’ comparisons between the experimental data and the theoretical XRD pattern (Figure S3).

### 2.3 Magnetic and Magnetothermal Properties

**Figure 4:**
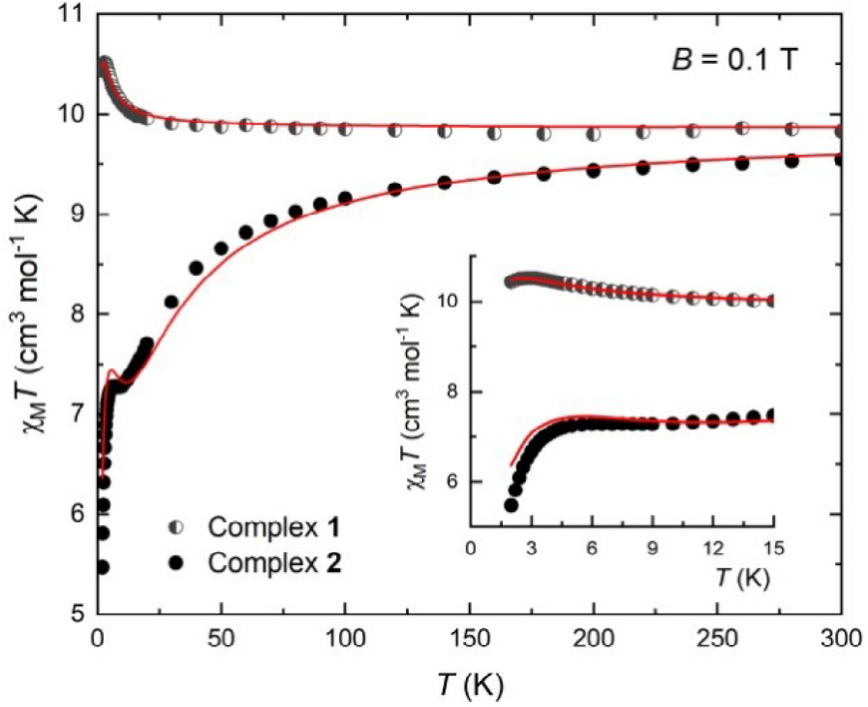
Temperature dependence of the *χ*_M_ *T* product for complexes **1** and **2**, under an applied magnetic field of *B* = 0.1 T. Inset: magnification of the low‐temperature region. Solid lines represent the fits (see text).

Direct current molar magnetic susceptibility, *χ*_M_, measurements for polycrystalline samples of **1** and **2** were carried out in the 2–300 K temperature range under an applied field of 0.1 T. The results are plotted as the *χ*_M_ *T* product versus temperature, *T*, in Figure 4. The *χ _m_* *T* values for **1** and **2** at room temperature are 9.84 and 9.58 cm^3^ mol⁻¹ K, respectively, very close to the theoretical value of 9.85 cm^3^ mol⁻¹ K, expected for two noninteracting Fe^III^ (*g*_Fe_ = 2.0) and one Ni^II^ ions (*g*_Ni_ = 2.1). For **1**, *χ*_M_ *T* remains stable until ∼ 30 K, below which it increases to a maximum value of 10.51 cm^3^ mol⁻¹ K at 3 K, before slightly dropping to 10.43 cm^3^ mol⁻¹ K at 2 K. This behavior is indicative of weak ferromagnetic interactions present in **1**, while the drop at the lowest temperature range may be attributed to intermolecular interactions and/or zero‐field splitting effects. For complex **2**, the *χ*_M_ *T* product remains constant until ∼140 K, below which it drops and levels off at ∼7.2 cm^3^ mol⁻¹ K in the 10 – 5 K temperature range, before reaching the minimum value of 5.47 cm^3^ mol⁻¹ K at 2 K. This behavior suggests the presence of dominant antiferromagnetic interactions in **2**. Indeed, the dominant ferromagnetic interactions in **1** and antiferromagnetic interactions in **2** were further supported by the *Curie‐Weiss* analysis for the 300–10 K temperature range, yielding a small positive *θ* value of +0.2 K for **1** and a negative *θ* value of ‐4.9 K for **2** (Figure S4). Furthermore, *ac* susceptibility measurements were carried out for both compounds. However, no out‐of‐phase signal was detected (not shown here), denoting the absence of spin dynamics.

**Figure 5:**
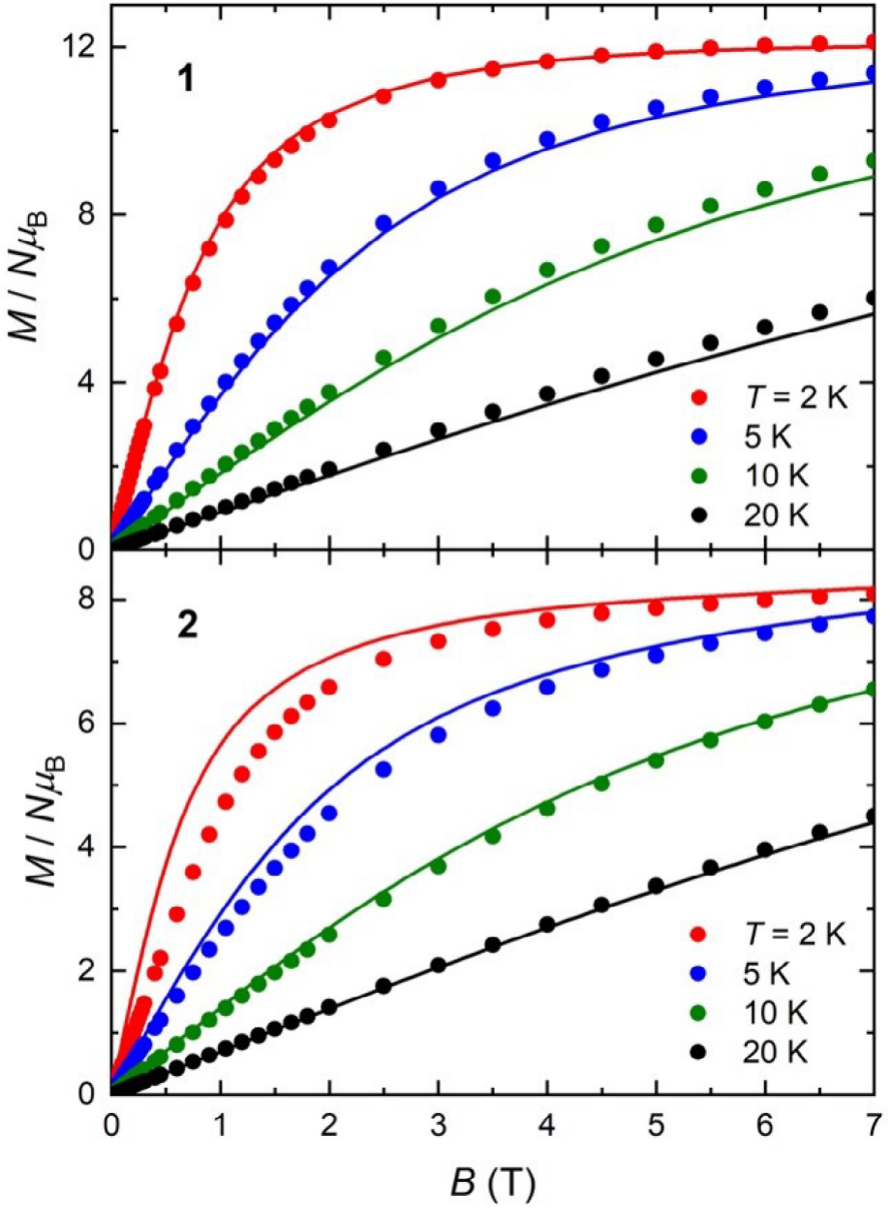
Isothermal molar magnetization *M* versus applied magnetic field data for **1** (top) and **2** (bottom) collected at *T* = 2, 5, 10, and 20 K. Solid lines represent the fits (see text).

Low‐temperature variable‐temperature and variable‐field magnetization, *M*, data were measured in the temperature range 2–20 K, in magnetic fields up to 7 T for complexes **1** and **2** (Figure 5). At the lowest temperature and highest field measured, *M* reaches a value of 12.1 and 8.1 *Nµ*_B_ for **1** and **2**, respectively.

**Figure 6:**
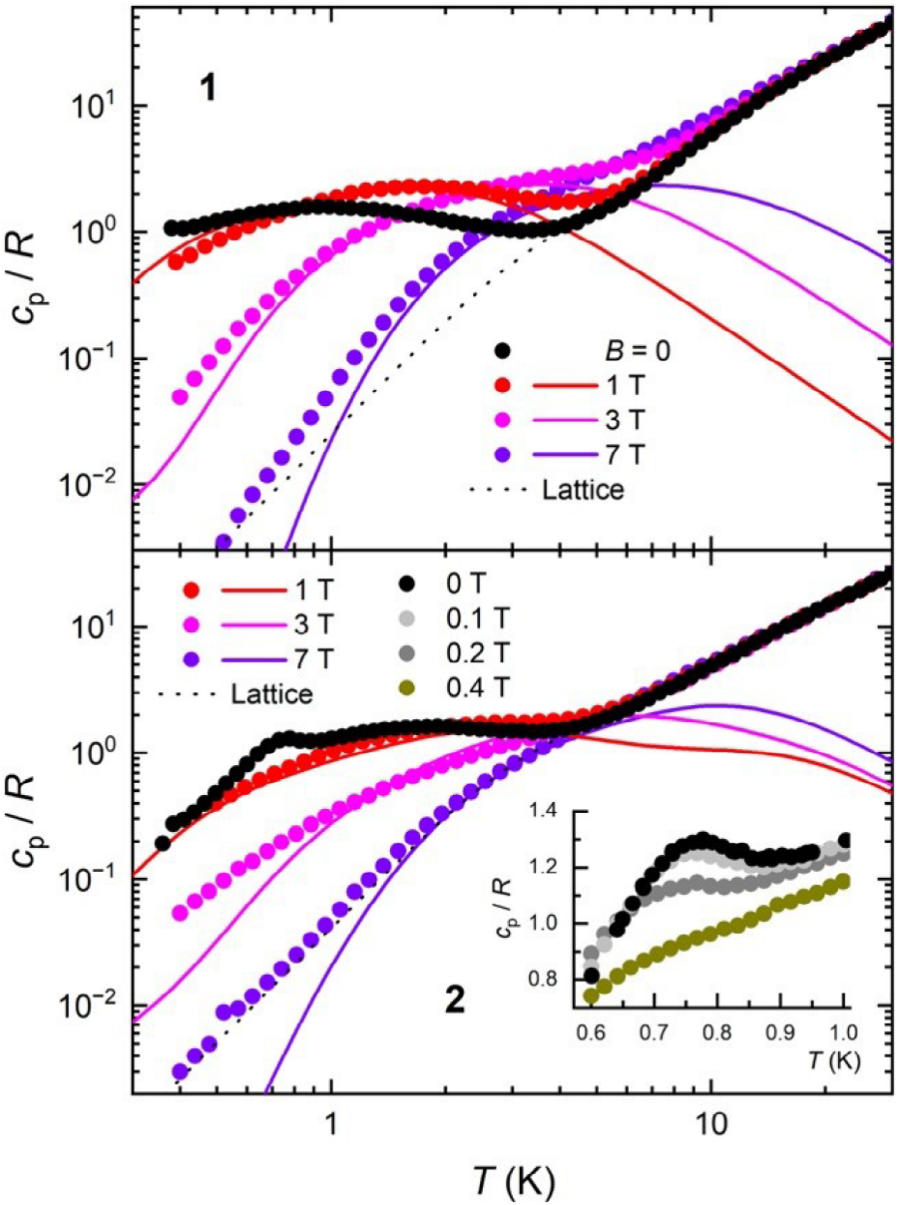
Heat capacity *c*_p_, normalized to the gas constant *R*, for selected magnetic field values for **1** (top) and **2** (bottom). Inset: magnification of the temperature range 0.6–1.0 K, highlighting the peak observed in the zero‐field data of **2** at 0.77 K, which indicates a phase transition to long‐range antiferromagnetic order. Lines represent simulations (see text).

Heat capacity, *c*_p_, measurements were conducted over the temperature range of 0.3–30 K and under magnetic fields *B* = 0, 1, 3, and 7 T for polycrystalline pressed pellet samples of **1** and **2**. Additional measurements for complex **2** were taken at *B* = 0.1, 0.2, and 0.4 T (Figure 6). For both compounds, the high‐temperature behavior of *c*_p_ is predominantly governed by the lattice contribution (dashed lines), which is effectively described by the Debye's law at low temperatures. Below ∼4 K, this relationship simplifies to *c*_p_/*R* = *aT* ^3^, with values of *a* = 2.5 × 10^−2^ and 4.1 × 10^−2^ K^−3^ for **1** and **2**, respectively. Notably, the heat capacity of **1** and **2** exhibits a strong dependence on the applied magnetic field at liquid‐helium temperatures. Each shows a Schottky‐like anomaly that shifts to higher *T* as *B* increases. Complex **2** reveals an additional feature, that is, a peak centered at *T*_N_ = 0.77 K in the zero‐field data, which arises on top of the Schottky‐like anomaly and is indicative of a magnetic phase transition. The peak is gradually suppressed with increasing magnetic field strength, vanishing at *B* = 0.4 T, thereby confirming its magnetic origin. The minimal impact of the lowest applied field, where the peak merely shifts to 0.76 K for *B* = 0.1 T, suggests that the ordering is likely antiferromagnetic.

To model the magnetic and magnetothermal properties of complex **1**, we employed the spin‐Hamiltonian $H=-\;J\sum {\vec{s}}_{i}\cdot {\vec{s}}_{j}-Ds_{z}^{2}+\mu_{B}\sum g_{i}Bs_{i}$, where $\vec{s}$ represents the spin operator, *J* is the pairwise exchange coupling constant for the Fe‐Ni interaction mediated by one *syn, anti η* ^1^:*η* ^1^:*µ* carboxylate group, and *D* is the uniaxial zero‐field anisotropy term, assumed to be nonzero only for the Ni^II^ ions. The fitting of the experimental data was conducted using the software PHI,^[^ ^7^ ^]^ which accommodates mean‐field intermolecular interactions via a “*zJ*” correction parameter. The best‐fit parameters obtained were *J*/*k*_B_ = +0.40 K, *D*/*k*_B_ = +4.3 K, *g*_Fe_ = 2.0, *g*_Ni_ = 2.1, and *zJ*/*k*_B_ = −7 × 10^−3^ K, thus confirming the presence of a dominant ferromagnetic interaction alongside very weak antiferromagnetic intermolecular correlations. The resulting calculated curves, depicted as solid lines in Figures 4, 5, and 6, show satisfactory agreement with experimental data.

Despite the structure of complex **2** suggesting a potential magnetic behavior as a spin chain system, our modeling indicates otherwise. This is also supported by the experimental zero‐field *c*_p_ (Figure 6), which lacks the low and broad anomaly characteristic of 1D magnetic correlations at high temperatures.^[^ ^8^ ^]^ Instead, the zero‐field *c*_p_ exhibits a high‐*T* Schottky‐like anomaly akin to that observed in nonzero field measurements. Moreover, the *χ*_M_ *T* data suggest stronger interactions in **2**, leading us to tentatively interpret its properties as resulting mainly from antiferromagnetic coupling facilitated by the *anti, anti η* ^1^:*η* ^1^:*µ* carboxylate bonding that forms the backbone of the coordination polymer. This results in antiferromagnetic {Fe^III^_2_Ni^II^} units that are intercorrelated by the weaker magnetic interactions present in **1**. Accordingly, we successfully applied the same model used for **1**, yielding the solid lines in Figures 4, 5, and 6 based on the best‐fit parameters obtained, namely, *J*/*k*_B_ = −5.76 K, *D*/*k*_B_ = +25.3 K, *g*_Fe_ = 2.0, *g*_Ni_ = 2.1, and *zJ*/*k*_B_ = −4 × 10^−2^ K. As anticipated, the agreement with experimental data is less satisfactory at the lowest temperatures and applied fields. Notably, this model cannot predict the phase transition, and the simple *zJ* term does not differentiate between intra‐ and inter‐chain interactions. The significantly larger *D* in **2** could be ascribed to the axis perpendicular to the basal plane of the octahedral Ni(II) being more tilted by ∼3.5° in **2** than in **1**. The *J* values obtained for **1** and **2** are in good agreement with previously reported heterometallic Fe^III^/Ni^II^ clusters (Table 1).

**Table 1:** Magnetic properties of representative Fe^III^/Ni^II^ clusters.

Formula	*J* [K]	*g*	Ref.
[(pzTp)Fe^III^(CN)_3_]_4_[Ni^II^L]_4_[OTf]_4_ **^.^** 10DMF**^.^** Et_2_O^[^ ^a^ ^]^	+9.5	2.2	[9]
[(Tp)Fe^III^(CN)_3_Ni^II^(tren)]_2_(ClO_4_)_2_·2H_2_O	+6.5	2.2	[10]
[{Ni^II^(teta)}{Fe^III^(phen)(CN)_4_}_2_]**^.^** 4CH_3_OH	+1.6	2.1	[11]
[Fe^ΙΙΙ^_2_Ni^ΙΙ^(L_1_)_4_(MeOH)_4_]	+0.40	2.1	This work
[Fe^III^_8_Ni^II^_6_L_24_(SCN_11_)Cl]	−0.04	2.0	[12]
[Fe^III^Cl_4_]⊂ Ni^II^_4_L_6_](OTf)_7_^[^ ^b^ ^]^	−0.09	2.1^*^	[13]
[Fe^III^Br_4_]⊂ Ni^II^_4_L_6_](OTf)_7_^[^ ^b^ ^]^	−0.12	2.1^*^	[13]
[{(NO)Fe^III^(bme‐dach)}_2_Ni^II^]^2+^ **^.^** 2BF_4_^−[^ ^c^ ^]^	−4.3	2.0	[14]
{[Fe^ΙΙΙ^_2_Ni^ΙΙ^(L_2_)_4_(MeOH)_2_]_n_}	−5.8	2.1	This work

^[a]^ L = 2,2,2‐tris(pyrazolyl)ethanol.

^[b]^ L = quaterpyridine.

^[c]^ bme‐dach = N,N‐bis(2‐mercaptoethyl)‐1,4‐diazacycloheptane.

* g corresponds to g_Ni_

## 3 Conclusion

In conclusion, in this work we report the synthesis, characterization, and magnetic properties of two closely related heterometallic Fe^III^/Ni^II^ species, upon employment of amino acid Schiff‐base ligands; a trimetallic [Fe^III^_2_Ni^II^(L_1_)_4_(MeOH)_4_] (**1**) cluster and a quasi 1‐D {[Fe^III^_2_Ni^II^(L_2_)_4_(MeOH)_2_]_n_} (**2**) chain (H_2_L_1_, H_2_L_2_: the Schiff‐base ligand derived from the condensation of 2‐hydroxynaphthaldehyde and gly (for **1**), or mAla (for **2**)). Both species present striking structural similarities, yet they display different magnetic behavior. Complex **1** displays dominant ferromagnetic interactions, while complex **2** shows antiferromagnetic behavior. We believe that this work represents a fine example of how subtle structural changes on a ligand can lead to drastic effects on the identity of the isolated species (discrete clusters vs. coordination polymers), and their magnetic behavior (ferro‐ vs. antiferromagnetic). More work is currently underway in order to test our hypothesis upon employment of different amino acid ligands, as well as different metallic pairs (3*d*‐3*d’*, or 3*d*‐4*f*).

## Supporting Information

Synthetic and crystallographic details, EDS spectra, pXRD diagrams and Curie‐Weiss analysis for **1** and **2** are included in the Supporting Information.

## Conflict of Interest

The authors declare no conflict of interest.

## Supporting information



Supporting Information

## Data Availability

Deposition Number(s) 2423115 (1) and 2423116 (2) contain the supplementary crystallographic data for this paper. These data are provided free of charge by the joint Cambridge Crystallographic Data Centre and Fachinformationszentrum Karlsruhe Access Structures service. Magnetic and magnetothermal data for compounds 1 and 2 can be retrieved from DIGITAL.CSIC at https://doi.org/10.20350/digitalCSIC/17141.
