# Supplementary material for: A Linear {FeIII 2NiII} Cluster, a Structurally Closely Related Quasi 1D {FeIII 2NiII} Chain
Source: Chemistry. 2025 May 28;31(36):e202500911. doi: 10.1002/chem.202500911 (PMC12202841; doi:10.1002/chem.202500911)
Supplement: Supplementary file 1 — Supporting Information [file CHEM-31-e202500911-s001.docx]

Chemistry – A European Journal

Supporting Information

**A linear {Fe^III^_2_Ni^II^} cluster and a structurally closely related *quasi* one-dimensional {Fe^III^_2_Ni^II^} chain**

Emmanouil K. Charkiolakis, David Gracia, Marc Ubach I Cervera, Marco Evangelisti* and Constantinos J. Milios*

**General**

All chemicals were obtained from commercial suppliers (Sigma-Aldrich) and were used without further purification/treatment. Powder XRD measurements were collected on freshly prepared samples of **1** and **2** on a PANanalytical X’Pert Pro MPD diffractometer. Magnetisation data were collected on a MPMS3 SQUID magnetometer equipped with a 7 T magnet. Diamagnetic corrections were applied using Pascal's constants. Heat capacity measurements were carried out on a PPMS equipped with a 9 T magnet and a ^3^He cryostat, using a thin pressed pellet (ca. 1 mg) of polycrystalline samples of **1** and **2**, thermalized by ca. 0.2 mg of Apiezon N grease, whose contribution was subtracted using a phenomenological expression. EDS measurements were performed on a JEOL Scanning Electron Microscope. Single crystal X-ray diffraction data were collected on a Bruker D8 VENTURE diffractometer (University of Crete), equipped with a PHOTION II CPAD detector. Data collection parameters and structure solution and refinement details are listed in Table S1. Full details can be found in the CIF files with CCDC reference numbers 2423115 and 2423116, for **1** and **2**, respectively.

**Syntheses of the complexes**

Synthesis of **1**

FeCl_3_·6H_2_O (0.135 g, 0.5 mmol), NiCl_2_·6H_2_O (0.059 g, 0.25 mmol), 2-OH-1-naphthaldehyde (0.172 g, 1.0 mmol), glycine (0.075 g, 1.0 mmol) and CH_3_ONa (0.028 g, 0.5 mmol) were added in 15 ml of MeOH and the solution was transferred to a Teflon-lined autoclave, and the autoclave was placed in an oven at 120°C for 12 hours. After cooling slowly to room temperature, dark brown crystals of **1** were obtained at a yield of ~70%, and collected filtration under vacuum, washed with a small amount of MeOH/Et_2_O (2 x 5 ml) and allowed to dry. Elemental analysis (%) calcd for C_56_H_52_Fe_2_N_4_NiO_16_ (**1**): C 55.71, H 4.34, N 4.64; found: C 55.62, H 4.19, N 4.51.

Synthesis of **2**

Complex **2** was synthesized in an analogous manner to **1**, upon using methyl-alanine (0.103 g, 1.0 mmol) instead of glycine. Dark brown crystals of **2** were obtained at a yield of ~60%. Elemental analysis (%) calcd for C_62_H_60_Fe_2_N_4_NiO_14_ (**2**): C 59.31, H 4.82, N 4.46; found: C 59.20, H 4.68, N 4.34.

**Table S1**. Crystal data and structure refinement for complexes **1** and **2**

|  | **1** | **2** |
| --- | --- | --- |
| Empirical formula | C_56_H_52_Fe_2_N_4_NiO_16_ | C_62_H_60_Fe_2_N_4_NiO_14_ |
| Formula weight | 1207.42 | 1255.55 |
| Temperature/K | 210 | 202 |
| Crystal system | Monοclinic | Triclinic |
| Space group | P2_1_/c | P-1 |
| *a*/Å | 12.207 (4) | 8.834 (5) |
| *b*/Å | 15.524 (3) | 13.596 (6) |
| *c*/Å | 11.6786 (14) | 13.644 (3) |
| *α*/° | 90 | 118.011 (12) |
| *β*/° | 110.247 (10) | 91.342 (18) |
| *γ*/° | 90 | 103.48 (2) |
| Volume/Å^3^ | 2586.7 (9) | 1390.1 (10) |
| *Z* | 2 | 1 |
| *ρ*_calc_ g/cm^3^ | 1.550 | 1.500 |
| *μ*/mm^‑1^ | 5.519 | 5.131 |
| *F*(000) | 1248.0 | 652 |
| Crystal size/mm^3^ | 0.30 x 0.32 x 0.25 | 0.33 x 0.31 x 0.27 |
| Radiation | CuKα (λ= 1.54178) | CuKα (λ= 1.54178) |
| 2Θ range for data collection/° | 6.2 to 137.2 | 7.4 to 137.5 |
| Index ranges | -18 ≤ h ≤ 18, -18 ≤ k ≤ 18, -14 ≤ l ≤ 13 | -10 ≤ h ≤ 10, -16 ≤ k ≤ 16, -14 ≤ l ≤ 16 |
| Reflections collected | 21900 | 18817 |
| Independent reflections | 4670 | 5101 |
| Data/restraints/parameters | 4345/0/362 | 4974/0/382 |
| Goodness-of-fit on F^2^ | 1.034 | 1.057 |
| Final *R* indexes [*I*>=2σ (I)] | R_1_ = 0.041, wR_2_ = 0.114 | R_1_ = 0.028, wR_2_ = 0.078 |
| Largest diff. peak/hole / e Å^-3^ | 0.97/-0.61 | 0.44/-0.45 |


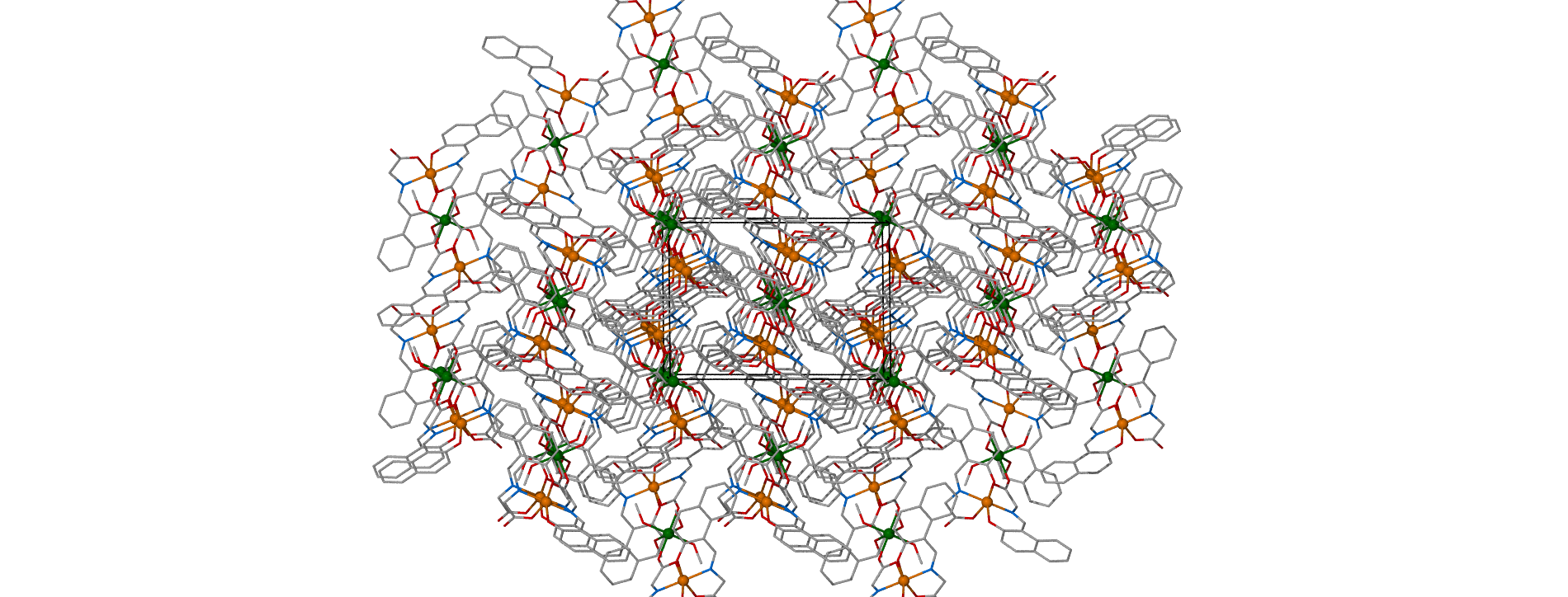

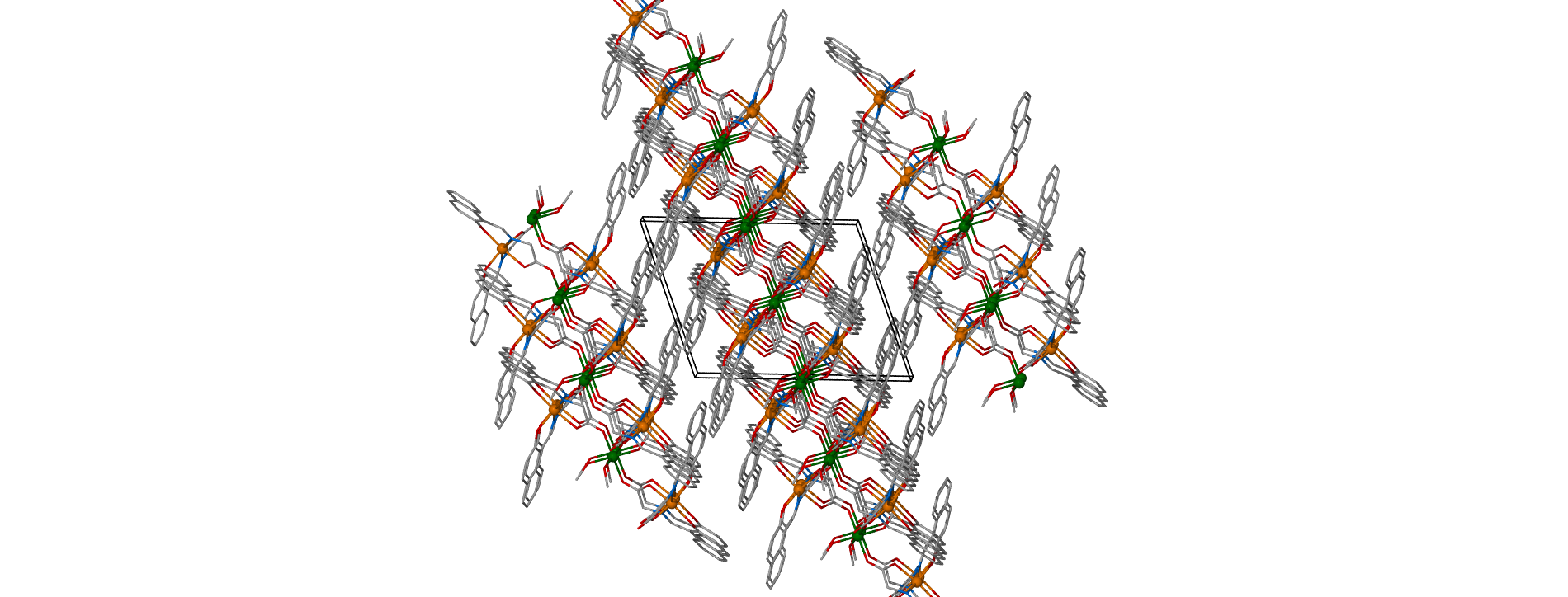


Fig. S1. Crystal packing for complex **1** viewed along the *a* axis (top) and *b* axis (bottom). Color code: Fe^III^ = orange, Ni^II^ = green, O = red, N = blue, C = grey.


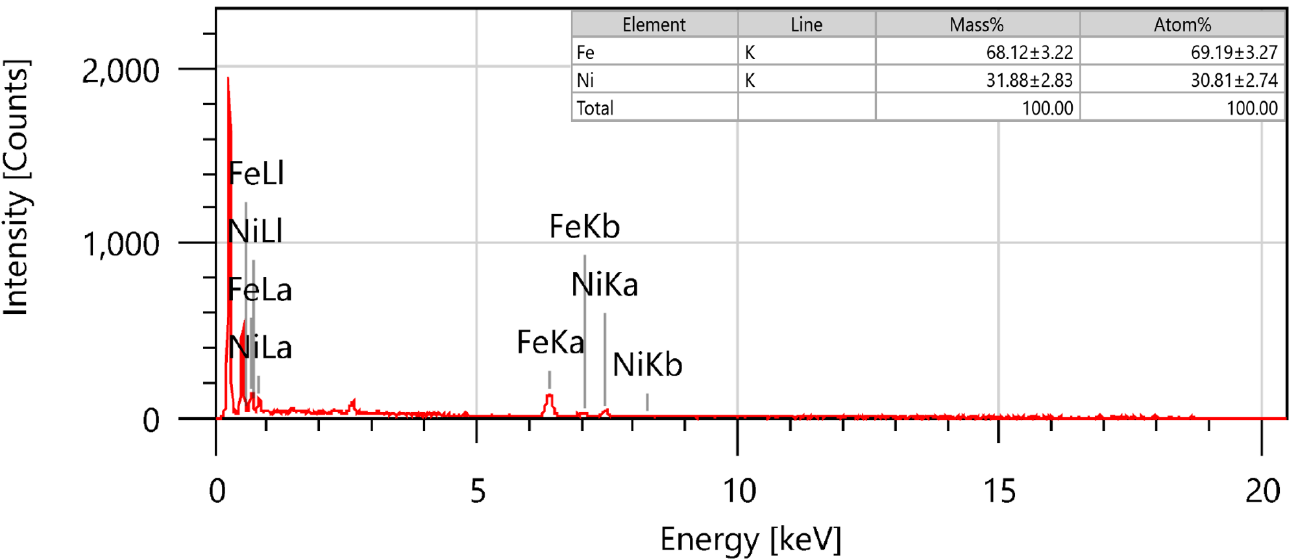

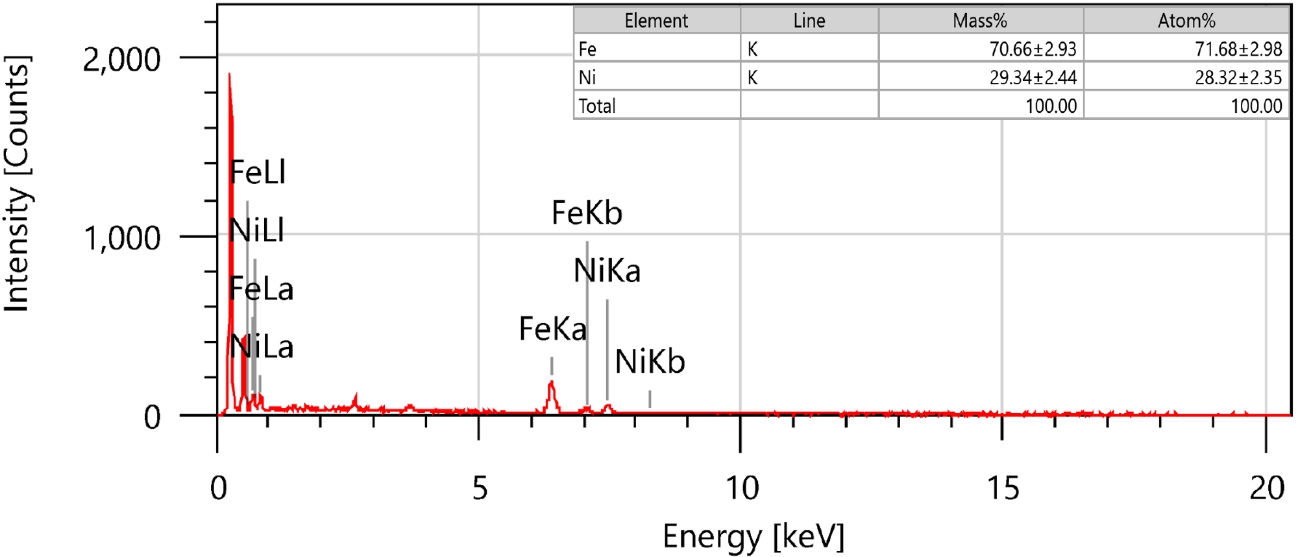


Fig. S2. EDS patterns for complex **1** (top) and **2** (bottom).


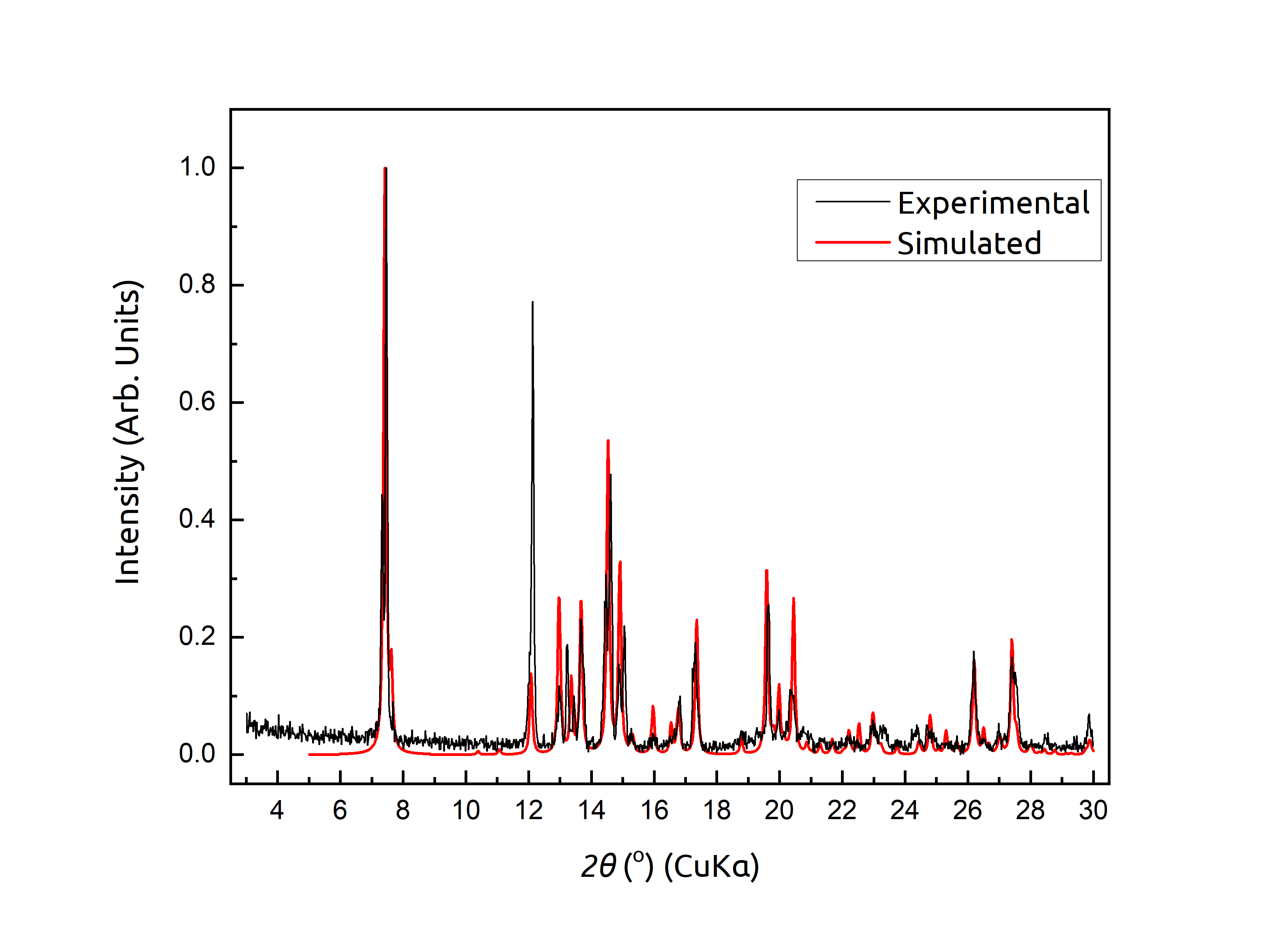

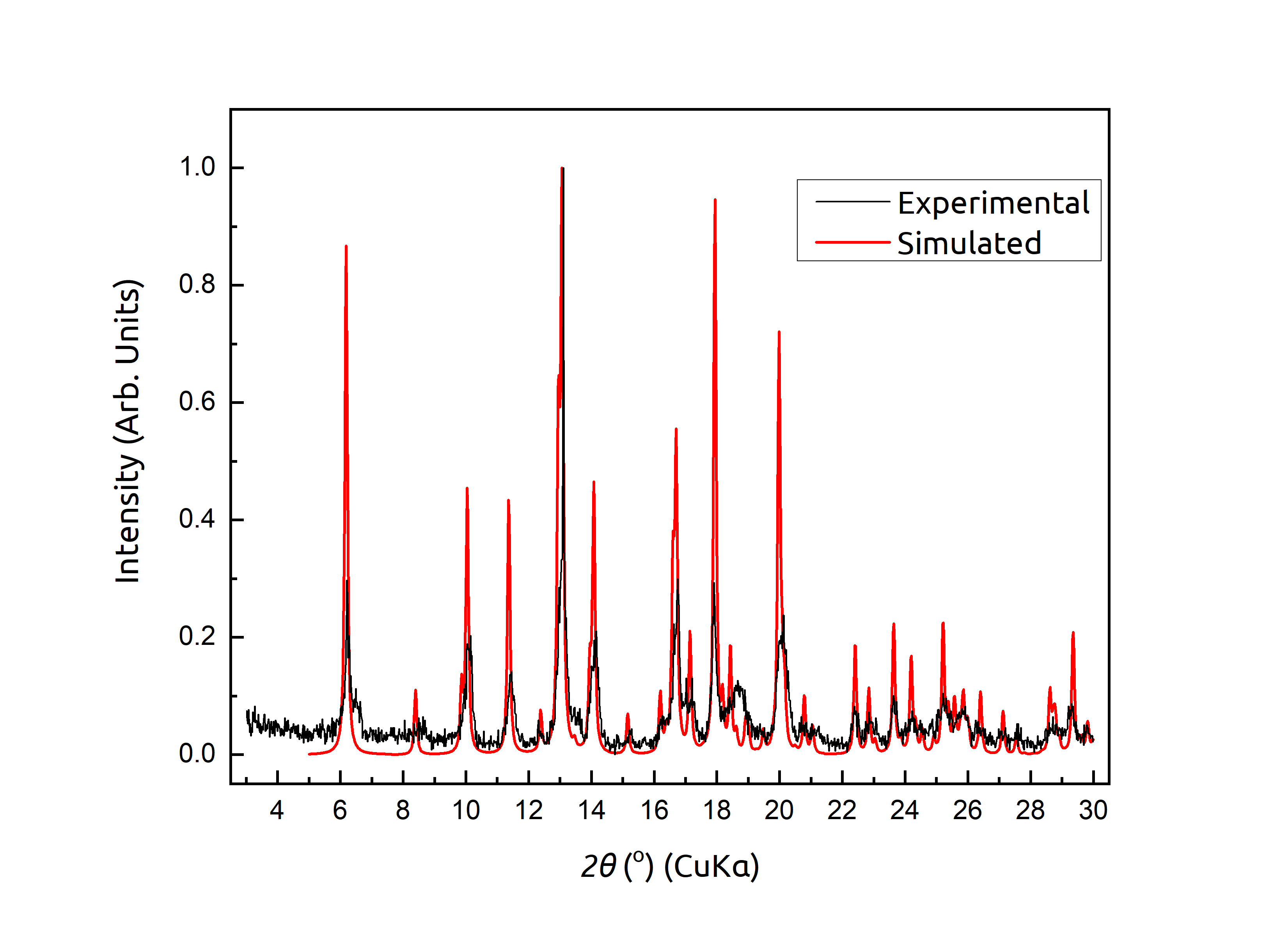


Fig. S3. Powder XRD pattern comparison for **1** (top) and **2** (bottom) and their simulated PXRD patterns.


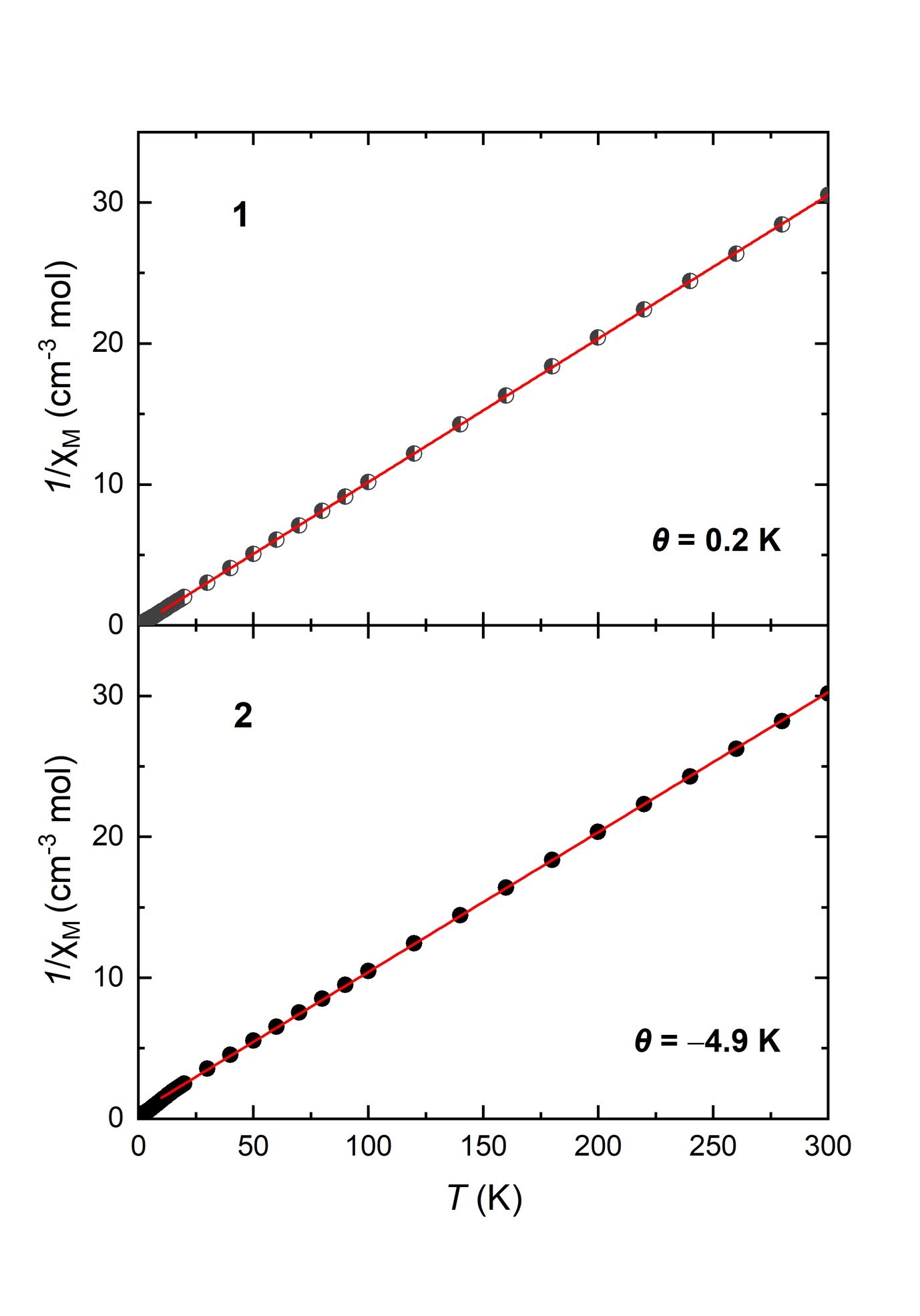


Fig. S4. Curie-Weiss fits for **1** (top) and **2** (bottom) in the 300 – 10 K temperature range.
